# Ethical and legal considerations in social media research for health technology assessment: conclusions from a scoping review

**DOI:** 10.1017/S0266462323000399

**Published:** 2023-10-16

**Authors:** Anke-Peggy Holtorf, Andriy Danyliv, Annekatrin Krause, Alissa Hanna, Yvette Venable, T. Joseph Mattingly, Li-Ying Huang, Miranda Pierre, Aline Silveira Silva, Donna Walsh

**Affiliations:** 1 PCIG Project Coordinator, Health Outcomes Strategies GmbH, Basel, Switzerland; 2 Novartis Pharma AG, Basel, Switzerland; 3 Patient Advocacy Europe, Novartis, Basel, Switzerland; 4 Patient Engagement, Edwards Lifesciences, Irvine, CA, USA; 5 Patient Engagement, ICER, Boston, MA, USA; 6 College of Pharmacy, University of Utah, Salt Lake City, UT 84112, USA; 7 Division of Health Technology Assessment, Center for Drug Evaluation, Taipeh, Taiwan; 8 Scottish Medicines Consortium, Healthcare Improvement Scotland, Glasgow, Scotland; 9 Patient Partner, Patient Voices Network, Vancouver, Canada; 10 European Federation of Neurological Associations, Brussels, Belgium

**Keywords:** social media research, social media listening, ethics, privacy, health technology assessment

## Abstract

**Objectives:**

The objective was to identify and describe the published guidance and current academic discourse of ethical issues and standards related to the use of *Social Media Research* for generating patient insights for the use by health technology assessment (HTA) or health policy decisions.

**Methods:**

A scoping review of the literature was conducted in PubMed and Embase and identified 935 potential references published between January 2017 and June 2021. After title and abstract screening by three reviewers, 40 publications were included, the relevant information was extracted and data were collected in a mind map, which was then used to structure the output of the review.

**Results:**

*Social Media Research* may reveal new insights of relevance to HTA or health policies into patient needs, patient experiences, or patient behaviors. However, the research approaches, methods, data use, interpretation, and communication may expose those who post the data in social media channels to risks and potential harms relating to privacy, anonymity/confidentiality, authenticity, context, and rapidly changing technologies.

**Conclusions:**

An actively engaged approach to ensuring ethical innocuousness is recommended that carefully follows best practices throughout planning, conduct, and communication of the research. Throughout the process and as a follow-up, there should be a discourse with the ethical experts to maximally protect the current and future users of social media, to support their trust in the research, and to advance the knowledge in parallel to the advancement of the media themselves, the technologies, and the research tools.

## Introduction/background

The use of robust research into patients’ needs, preferences, and experiences, as well as patient participation are the recommended approaches to ensure that the patient perspective is included in value determination in the health technology assessment (HTA). Increasingly, communication of patients and caregivers on social media (highly interactive mobile or web-based platforms through which individuals or communities share, co-create, discuss, and modify user-generated content) is used by patient organizations, industry, or other stakeholders as a source for such research. In relation to HTA, the findings may support the early scientific advice interactions or as part of the submission dossiers to both regulatory and HTA agencies for approval purposes.

The benefits of using *Social Media Research* for identifying patients’ needs, experiences, and preferences can be related to the unsolicited and open nature of the shared information, as well as the broad coverage of the patient population as compared to individual input or focus groups. However, *Social Media Research* is prone to certain risks and biases due to the personal nature of the information shared on social media. Particular sensitivity relates to ethical and legal aspects concerning the privacy and protection of the people who communicate and of other people they may communicate about (1). Currently, there is a lack of guidance on how to conduct *Social Media Research* appropriately to meet the ethical standards and the legal requirements of privacy protection as required for supporting HTA or healthcare policy decisions. This gap may be due to the innovation frequency of the communication media, the different ways of how they are used by all stakeholders and the research methods applied to analyze them. The media used by individuals to share views and to publish material online, the search engines available to users, and the approaches for conducting research with these communications are constantly evolving.

On the other hand, *Social Media Research* is conducted by a variety of disciplines that may not yet adhere to common guidance or “best practices,” including sociology, computer science, media/communications studies, public health research, and allied fields, but also others such as market research, epidemiology, anthropology, or bioethics. The varied aims and perspectives lead to high diversity in ethical standards which are applied across studies on all levels of the research ecosystem: among researchers, research groups, research entities, review boards, funding councils, publishing bodies, and nations (1).

Together with the increase of research on social media, examples of misuse of these data are surfacing. This triggers actions such as increased control for access to closed group on proprietary sites, restrictions for data sharing, closed access to data only within proprietary systems precluding use by external or independent researchers (2). These measures strengthen data privacy protection but may hinder the conduct of *Social Media Research*.

A consensus on the requirements to be met in the planning and conduct of *Social Media Research* to achieve a high level of protection for the users of social media while enabling *Social Media Research* in a way that it benefits the patients and healthcare systems would help researchers and users of the research results in improving or assessing the quality of the research. The aim of this report is to summarize the current academic discourse of ethical issues and standards, and to advance this discussion and guidance in order to foster the robustness and integrity of Social Media Research for informing regulatory, HTA or health policy decisions.

## Methods

A literature search was conducted in PubMed and Embase with the following search strategy: (Research OR Listening OR Mining) AND (Social Media) AND (Legal OR Privacy OR Ethical OR Ethics). Non-relevant publications were eliminated in three steps: (i) a title screen; (ii) classification of remaining publications by three reviewers as “include,” “exclude,” or “potential” based on the following criteria (a) containing information relating to ethical, privacy or legal issues related to research in social media and (b) related or relevant to the use of the research for informing regulatory or other assessments of technologies or for healthcare policy decisions; and (iii) conflicts in classification were resolved by discussion. Each reviewer reviewed and summarized the findings of one-third of the articles classified as “include” or “potential” and made a final decision for inclusion.

The occurring themes were grouped by themes of ethical and privacy-related challenges, or by reported principles and guidance on the conduct of the *Social Media Research*. The occurring themes were mapped into the relevant items on a mapping software (MIRO). In this way, the three researchers were able to enrich existing or create new themes and substantiate them with the text from the publications. A summary of all extracted information including all references is available in the online supplementary material (Supplementary file 1).

The main challenges as reported in the scientific literature were summarized together with the proposed measures for mitigation. The identified themes and some of the strategies proposed in the literature were further developed through classification of the privacy and ethical aspects to propose a list of principles for the conduct of *Social Media Research*, and to provide a short checklist of the critical considerations to mitigate specific privacy and ethical risks.

## Results

**Figure 1.**
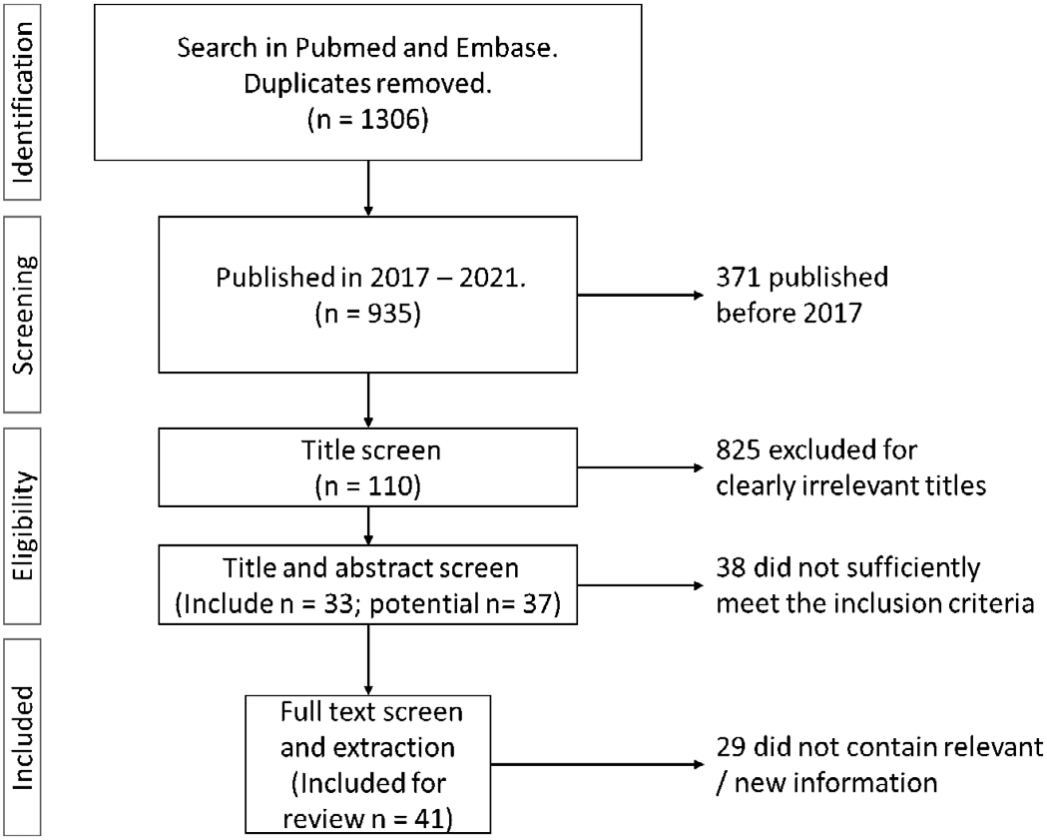
PRISMA diagram for the scoping review.

The process of the scoping review is reflected in the PRISMA (Preferred Reporting Items for Systematic Reviews and Meta-Analyses) diagram in Figure 1. Of the 1306 references retrieved in the general literature search, 935 were published between 2017 and April 2021. The first title screen excluded 825 references. Of the remaining 110 references, 38 were classified as “Exclude,” 33 as “Include” and 38 as “potential.” Finally, based on the full-text articles, the extracts of 41 publications were used in the thematic analysis. The main themes presented in this report are (i) definitions of social media research, (ii) ethical aspects, (iii) privacy, (iv) informed consent, (v) anonymity and confidentiality, (vi) authenticity and justice, (vii) beneficence and non-maleficence, (viii) context, and (ix) ethical review.

### Definition of social media research

For the purposes of this report, *Social Media Research* is defined as research with data originating from any social media platforms including, but not limited to Twitter, Facebook, YouTube, Instagram, Reddit, blogs, and chatrooms or forums (closed/password-protected and open/non-password-protected). As the method of research, this may refer to large quantitative data mining/modeling methods through to more qualitative in-depth analyses. The aims of *Social Media Research* generally are to reveal new insights into information sharing or policy discussions, to understand personal experiences and opinions or sentiments from the individual or patient community perspective, to conduct epidemiological studies, to identify and detect adverse events or other clinically relevant reports, or to observe online behavior in general.

### Ethical aspects

Due to the fluidity of uses and content within online settings, existing guidelines such as the ones produced by the Association of Internet Researchers (AoIR) advise a deliberative, “bottom-up” approach to research ethics that allows for differing disciplines and research contexts, as opposed to providing a “top-down,” universal set of principles and regulations. Researchers are advised to engage in a deliberative process when making ethical decisions about online research, taking into account the vulnerability of online data and balancing the rights of authors (who might be considered “communities,” “authors,” or “participants”) against the potential benefit of the research (3).

Privacy, anonymity and confidentiality, authenticity, and (rapidly changing) technologies for communication, data collection, and analysis have been identified as key themes in the ethical appraisal of *Social Media Research* (4).

In addition, to improve consistency, quality, and integrity of ethical assessments over time, a requirement for a review by research ethics committees for all social media research has been suggested by Samuel and coauthors – even if it often may be declared exempt in the ethical review process (1).

### Privacy

Privacy has been declared a human right by the United Nations. Privacy means “freedom from unauthorized intrusion” (https://www.merriam-webster.com/dictionary/privacy) and, in more detail, “freedom from damaging publicity, public scrutiny, secret surveillance, or unauthorized disclosure of one’s personal data or information, as by a government, corporation, or individual” (https://www.dictionary.com/browse/privacy).

What is authorized in social media, is usually described in the privacy settings, which generally are constantly refined through resetting of user-chosen settings, leaving users resigned under the insurmountable task to understand them, maintain control, and ensure the protection of their own data. In addition, hidden in the jungle of legal text, service providers often secure the right for tracking and reusing the material at their own liberty. As Hunter and coauthors point out, “… public health researchers must recognize the self-serving interest of Social Media corporations and work within ethical principles that protect individual users, who are often powerless and uninformed in the labyrinthian Social Media environment” (4).

Therefore, researchers and ethics review committees need to be guided and trained to minimize the risk to any of the people included or concerned by the research.

### Informed consent

In clinical research, human subjects voluntarily confirm that their data can be used in a particular study, after having been informed of all aspects of the trial that are relevant to the subject’s decision to participate. However, the need for such consent in *Social Media Research* is debated and there is a controversy between *data manifested public* by the posts’ authors and the *need for consent for extraction and processing* (2–5;6).

Collecting the consent retrospectively for thousands of posts from a large number of individuals may not be feasible, and in addition, the consent may be implied in the terms and conditions of the use of platforms or by the public nature of the posts (6;7). The dilemma is that the use may be legal according to the user terms but not legitimate according to “human rights.” Patients posting data on publicly accessible social media are not always comfortable with these data to be used for specific research purposes, and generally, users do not pay attention or do not understand the implications of the terms of use (4–8).

### Anonymity and confidentiality

Data processing, security, and management must respect the applicable data protection legislation (national/local plus wider legislation, such as on European level, where applicable) (4;5).

In responsible social media research, users and third parties they may communicate about (e.g., parents communicating about the health of their children) must be protected against any potential risk for re-identification and against intrusion into their personal spheres (10). A special challenge with *Social Media Research* is that the never forgetting and highly networked characteristic of the internet limits the effectiveness of traditional methods of anonymization and hence, users may often not be sufficiently protected against reidentification. (4–9). For example, special risk of re-identification may occur in the space of rare diseases where only a small number of patients with unique combination of characteristics are active (11).

Several measures can help to minimize the risk of identification. Firstly, only necessary data is collected, and any identifiable information is excluded. Secondly, data access permission policies as well as computer infrastructure cater for the safe use of data and prevent data loss or disclosure. Finally, reporting abstains from including any identifiable information. For example, quotes are frequently used in qualitative research. However, direct quotes may easily allow re-identification through a simple search tool. One way to reduce the risk of re-identification of individuals can be to distance the analysis from the point of data collection by using intermediaries (data/analysis brokers) (9).

### Authenticity and justice

If Social Media Research is used to reveal matters relevant to the patients, healthcare decision makers must be able to trust that the conversation used for the research comes directly from the patients of interest (authenticity), that the breadth of patient population of interest is well represented by the research (representation), and the principle of justice is fulfilled. The principle of justice includes appropriate and fair participant selection, equal access to the research, and sharing in the benefits realized from the study findings (13).

Fake accounts, automated bots, or “astroturfing” (individuals adopt false identities and establish a false sense of group consensus or grassroot movements) are examples of how masses of Social Media communication are created that do not originate from the target population of the research (4) and thus, are neither authentic nor representative. Thought must also be given to potential bias (4–12) and the risk of overreach (14), that is the inclusion of data of people who are not in the target population of the study.

The lack of authenticity, justice and representation can impede the relevance of the results. Due to the uncertainty around some of the requirements relating to authenticity, justice, and representativeness, the interpretative value of the information resulting from social media research needs to be verified and corroborated by other means (e.g., medical data, patient interviews/focus groups) (12).

### Beneficence and non-maleficence

The primary goal of any social media study should be beneficence, meaning the welfare of the participating subjects, and the avoidance of maleficence, meaning anything that opposes the welfare of any research participant. Hence, any social media research should only proceed if more benefits than harms are expected (13) and measures have been introduced to maximize benefits and minimize harms (4). Key considerations in such assessments include (12): (a) How can the data be utilized for the common good whilst respecting individual rights and liberties? (b) What are the acceptable trade-offs between individual rights and the common good? (c) How do we determine the acceptability thresholds for such trade-offs?

### Context

In many cases of *Social Media Research*, most of the context of the communication will remain unknown as only certain parts of the communication are extracted and analyzed. When interpreting the data, the limitation of unknown context needs to be recognized, acknowledged, and its impact considered (15;16).

While context often is essential for precise understanding, revealing the contextual details may also increase the risk for identification. Hence, a balance must be found between which details of context are required for correct interpretation and which ones would expose individual subjects to the risk of re-identification.

### The need for ethical review

Currently, researchers and Research Ethical Committees (REC) or Institutional Review Boards (IRB) are often left to their own devices in the development of their own frameworks based on trial-and-error learning approaches in an environment of rapidly changing and developing methods (4). The formation of a flexible framework for things to consider and approaches to employ to maintain ethical conduct in public health research using social media data is recommended. Requirements may not be imposed by the IRB due to lack of expertise and guidance, but if they have potential ethical implications, they should still be considered by social media researchers (17). Throughout the research life cycle, it is important for IRB/REC members to engage and listen to the researchers and build on their expertise which may originate from relevant practical engagement (18).

Dedicated “technology ethics boards” (comprised of individuals with expertise in the technology as well as those versed in the ethical, legal, and social implications of data use) could be convened in universities and other research organizations to educate and advise scientists, research participants, IRBs, and the public (1–9;18;19). Such boards should represent a broad range of perspectives (research, sociology, patient organizations, social media communication, computer scientists/data experts, legal knowledge) to reflect the breadth of perspectives from which *Social Media Research* in healthcare is undertaken or to be reviewed (1–19). Reviewers are trained regularly on new developments in social media (19), they inform themselves on the special issues related to research in social media, and guidance is appropriately updated and available to them.

## Discussion and recommendations

With the intention of creating a simple guide not only for researchers and ethics reviewers, but also for HTA practitioners who use the results of *Social Media Research*, the findings of the scoping review were consolidated into a framework for future guidance tables (see Tables 1–3).

**Table 1.** Ethical and quality related aspects must be considered along the life-cycle of Social Media Research (adapted from (20))

	Planning, scoping	Data collection and analysis	Reporting and follow-up
Tools/methods	Selection: strength and weaknesses compared to alternatives	Application	Transparent reporting of methods and limitations
Data	Selection: strength and weaknesses compared to alternatives	Transparency	Transparent reporting including limitations
Risks	Assessments of possible risks and mitigation options	Active risk management	Follow-up risk assessment: Were all risks and threats foreseen? Would the consent of users change if they knew their data was being used for this research/intervention?
IRB/REC	Life-cycle approach along research (Planning, execution, reporting, follow-up) by qualified IRB/REC (specifically trained for technical and ethical challenges of social media research); repeated dialogue between IRB/REC and researchers		
	*Crowdsourced “Hippocratic Oath for Data Science”*: *Ensure that all data practitioners take responsibility for exercising ethical imagination in their work, including considering the implication of what came before and what may come after, and actively working to increase benefit and prevent harm to others.*		

Table 1 summarizes the guiding principles to consider in relation to the research tools, the data, and the ethical review process along the entire life cycle of *Social Media Research* form conceptualization and scoping to reporting and follow-up. For the tools and methods, the appropriateness, the reliability, and the replicability are of utmost importance. The research data must be representative and accessible to a broad range of the researched population, the required level of consent must be respected, and data should be sufficiently anonymized and safely managed and stored. The results need to be validated through other means and approaches. The ethics review process is not a one-time review and approval but happens along the lifecycle of the research throughout the planning of the research, the implementation of the study and the reporting, and is conducted by a specifically trained and up-to-date qualified review committee. Finally, a follow-up evaluation is performed to understand whether all risks and threats had been considered and whether the attitudes towards consent would change if the users had known that their data was used for this research.

In addition, Table 2 may serve as a more detailed checklist for the aspects related to research justification, research data and data sources, tools and methods, and legal requirements, which must be considered and reported in planning and reporting *Social Media Research*. While this checklist should be followed by researchers, it can also be used to document the appropriateness of the study for the potential use in HTA evidence appraisals.

**Table 2.** Principles for Social Media Research (adapted from (20–22) and others in this review)

Justification of research	Purpose of the use of the social media data	Are there other ways to address the research question and aims and is social media research the best option?
	Description of “public benefit” and “public interest”	Why is it justifiable to use private communication as data source for this research? (Balance between benefits and risks) Can the data be utilized for the common good whilst respecting individual rights and liberties?
Data	Choice of the social media data and reasons for choice	Are the chosen data sources representing the target population in the best way? Is the demand for justice (participant selection, access to research, sharing in the benefits) fulfilled?
	Type of social media content to be applied	What kind of data is used (text content, images, videos, blogs, vlogs, conversations, etc.)? How are the characteristics of the respective data sources accommodated for? Which level of consent is expected or required?
	Time period covered by the dataset	Is the time period relevant to research (incl. Timing of postings, duration, periodicity/ frequency, evolving content) and necessary
	Social media user demographics and specific population of interest	Which information can be gathered to ensure that the inclusion criteria are met? How big is the risk of too broad or too narrow inclusion?
	Generalizability, replicability	How is generalizability and replicability for the defined purpose addressed?
	Anonymization	Which methods are used for anonymization? Which risks are remaining for de-anonymization and how can they be mitigated?
	Adopted data management approach including data curation	How is the management and storage of data and consent defined and organized? Which methods for data retrieval are defined and applied (e.g., synonyms, provision for spelling variants/mistakes, lay language, etc.)?
Tools, methods	Appropriateness of Methods	Are there better methods around/is this the best method? Is it the right method to meet the goals of the research?
	Mitigation against any “skewing”/bias	Which methods are used to minimize the risk for skewing/bias through data selection or analysis? Which methods will be used to validate results?
	Methods of analysis	What are quantitative/qualitative research techniques? (dynamic approaches; algorithms applied across or specifically to the data sources)
Legal/ethical	Terms of use from the data provider	Does the research protocol meet the terms of use of the data source/provider?
	Principle of secondary use of data	Have interactions with the researched community been excluded by the protocol? Is secondary data use legally acceptable?
	Data protection legislation and ethical standards	Does the research method align with applicable data protection legislation (platform, geography, other)? Which ethical standards and considerations will be applied (e.g., Ethical Review process)?

Finally, Table 3 summarizes some actions or activities that have been clearly identified to alleviate the risk to those who communicate on social media or to those they may communicate about or on behalf of, or to the quality of the research itself. Therefore, these actions should be avoided. They include intrusion in private environments (e.g., closed communities), uninvited interaction with target users, verbatim quotes or individual demographics in the reporting, the use of historic and potentially outdated data or text, and finally, the assumption that a point review for ethics is sufficient.

**Table 3.** Preventive considerations of actions to be avoided (based on (23–25))

Intrusion in clearly private environments	Websites that require a log-in or password should not be used as this represents a private environment; therefore, individuals would not expect to be observed
Interaction with the target users	Do not influence the content posted on social media (e.g., posting targeted questions or adding own comments) without consent/transparency
Verbatim quotes	Do not use Verbatim quotes in reports/publications (risk of re-identification). Exception: with consent of contributor.
Individual demographics	Do not report any individual demographic data obtained (exception: if necessary to address the research aims)
Historic text	Do not use historical text in a non-contextual way (i.e., out of context). It may be maybe outdated, even from contributor perspective and the historical opt-out or consent is difficult/unlikely
Ethics assessment accomplished	Ethical appraisal is not a one-time assessment, but requires reassessment throughout the research life cycle

## Conclusions and recommendations for the use of social media research to inform HTA or health policy decision making

We have reviewed the existing guidance and current academic discourse of relevance related to the use of *Social Media Research* for generating patient insights that can inform HTA or health policy related decisions.

The main challenges reported in the publications were summarized together with the proposed measures for mitigation. Based on the findings of the scoping review in terms of the challenges and mitigation approaches, we propose in this report a set of practices and a checklist that can be applied when conducting *Social Media Research* to assure that the privacy and ethical risks are minimized. This aggregated list can be used for future guidance on the conduct of *Social Media Research* and for its use for decision making and should lay the base for further improvements in future based on the learnings resulting from systematic interaction between researchers and ethics experts as well as systematic follow-up.

*Social Media Research* has the promise to reveal new insights into patient needs, patient experiences, and, possibly, patient behaviors which could have high relevance to patient communities and give new directions for public health policies. However, the research approaches, methods, data interpretation, and communication may pose new challenges to researchers and ethics review boards in preventing potential harm to those who post the data on social media channels as well as to the people they are communicating about.

Although this report provides a set of principles and checklists on what to do and what to avoid when performing *Social Media Research*, it is strongly recommended to take an active and engaged approach to ensuring ethical innocuousness by carefully following best practices at each step of the research including the planning, the conduct, and the communication or dissemination. Throughout the process and as a follow-up, there should be a dialogue between the researchers and the ethics reviewers on a case-by-case basis to maximally protect the current and future users of social media, to support their trust in the research, and to advance the learnings in parallel to the advancement of the media themselves, the technologies, and the research tools.

## Supporting information

Holtorf et al. supplementary material Holtorf et al. supplementary material

## Supplementary material

The supplementary material for this article can be found at http://doi.org/10.1017/S0266462323000399.
