# Supplementary material for: Ethical and legal considerations in social media research for health technology assessment: conclusions from a scoping review
Source: Int J Technol Assess Health Care. 2023 Oct 16;39(1):e62. doi: 10.1017/S0266462323000399 (PMC11570170; doi:10.1017/S0266462323000399)
Supplement: Holtorf et al. supplementary material [file S0266462323000399sup001.docx]

Extracts for Good Ethics practices / Ethical Principles in Social Media Research from Published Literature

(Andrii Danyliv, Anke-Peggy Holtorf, Annekatrin Krause in Nov. 2021)

# Definition of Social Media Research

For the purposes of this report, Social Media Research (SMR) is defined as research with data originating from any social media networking site including, but not limited to: Twitter, Facebook, YouTube, Instagram, Reddit, blogs, and chatrooms or forums (closed / password-protected and open / non-password-protected). As method of research, this may refer to large quantitative data mining/modelling methods through to more qualitative in-depth analyses. The aim of SMR generally is to reveal new insights into information sharing, policy discussions, personal experiences and opinions or sentiments from the individual perspective, or to observe online behaviour in general [1].

----------

# Overview of literature on issues with SM

## Deliberative approach to ethics for social media research.

The media through which individuals publish material online, and the search facilities available to users, are constantly evolving. Due to the fluidity of uses and content within online settings, existing guidelines such as the ones produced by the Association of Internet Research (AoIR) advise a deliberative, “bottom-up” approach to research ethics that allows for differing disciplines and research contexts, as opposed to providing a “top-down,” universal set of principles and regulations. Researchers are advised to engage in a deliberative process when making ethical decisions about online research, taking into account the vulnerability of online data, and balance the rights of authors (who might be considered “communities,” “authors,” or “participants”) against the potential benefit of the research [2].

However, Samuel and Derrick [1] point out, that Health-related Social Media Research is likely to be particularly ethically sensitive due to the personal nature of the information shared, and the more obvious risks to personal identities and profiles.

On the other hand, this research can typically be done by a wide array of research disciplines including not only sociology, computer science, media/communications studies, public health research and allied fields, but also others such as market research, epidemiology, anthropology, or bioethics. With this high diversity and low level of ethical guidance, there is a strong risk of differing individual interpretations for which ethical approaches should be applied at each Ethics Ecosystem governance level - by different researchers, research groups, research institutions, funding councils, publishing bodies, and nations [1]. Thus, important ethical questions may be answered differently by different evaluators, and ethically problematic research may sometimes not be detected and prevented, and thus, the research subjects may be exposed to unnecessary risk to be harmed. They therefore propose for the UK, to require a review by research ethics committees – even if often declared exempted - for all social media research to arrive at a higher level of consistency and quality over time [1].

Examples of misuse of social media communication are accumulating and with this, social media platform / sites try to strengthen the (user-perceived) privacy, and hence, it is becoming increasingly difficult for researchers to access social media communication / data for their research. For traditional academic researchers, this is problematic as industry researchers control more and more of the research space through their proprietary systems [3].

---------------

# Potential research bias

In Social Media Research, there is a risk that there is no clear delineation between researcher and participant. For example, a study on patient perceptions by a patient organization, may include comments of the patient researchers themselves or of people they know, which may be problematic [4].

-----------

# Guidance

Numerous institutional and professional bodies have published guidelines or have developed case studies and/or fora to help scholars navigate the nuanced and complex ethical terrain of SM research (See *Reports & Guidance Summary*, page *13*).

------------

# Context

In the assessment of ethical issues, context is important. Context has many dimensions such as group or cultural context, the topic context, the methods applied (e.g., quantitative, qualitative), the research specialty, and the commercial context (platform’s ownership, infrastructure, and change of both) [5–6].

# Anonymity & Confidentiality

Anonymity of the people, whose communication content is used for the research is ethically essential. However, the never forgetting and highly networked characteristic of the internet may limit the success of the effort to protect the users and leave traditional methods of anonymization fruitless [7–8].

In addition, the content of the data (communication, images, videos etc.) may intentionally or unintendedly reflect back on other individuals (family, friends, children, or other social contacts) and reveal information on them or their health [9]. Even with consent of the primary user, this potential impact on secondary people is not resolved [7]. The risk to secondary people exists even with deidentified aggregate data from individuals can reveal information pertinent to others [10]

As another part of confidentiality considerations, there is the question how the research material is available to researchers, particularly with the increasing recognition of the values of the material, and the growing tendency of social media actors to restrict access to their data.

# Authenticity

Fake accounts, automated bots, or ‘astroturfing^[[1]](#footnote-1)^’ are examples how masses of SM communication are created that do not originate from the target population of the research. Although there are tools to detect the likelihood of bot communication, ‘astro-surfing’ (sponsored by parties with specific interests) are more difficult to identify and could strongly influence the study results of social media research [7].

The communication in social media only becomes valuable for public health after data analysis and interpretation. The interpretative value of the resulting information needs to be verified and corroborated with other means (e.g. medical data, patient interviews/focus groups) [11].

# Beneficence and Non-Maleficence

Under no circumstances can it be assumed that patients altruistically donate their clinical or self-generated data (social media, wearables etc.). A survey among patients in an emergency room, only half of the responders were willing to donate some of their data immediately and about 20percent were not willed to donate any data, even after death [12]. In addition, there was a high interest in knowing which data were used for what and what the findings were. In a survey with stakeholders for participatory health through social media the following concerns were mentioned as most important by most: data privacy, security, and quality assurance [13].

This challenges the assumption that consent is unnecessary [14] and shows how important it is to weigh benefits & Harms against each other. Key questions to ask for such assessment [11]:

- - How can big data be utilized for the common good whilst respecting individual rights and liberties?
  - What are the acceptable trade-offs between individual rights and the common good?
  - How do we determine the thresholds for such trade-offs?

The principle of beneficence involves weighing study risk and benefit to determine whether the research should proceed. In an era of data collection using pervasive sensing and ubiquitous computing technologies, we see increased sensitivities to privacy breach, data confidentiality, and all aspects of data management (e.g., collection, secure storage and sharing) which we mapped to the principle of beneficence (non-maleficence) (Nebkerer 2017)

- - referred as minimizing harm in (Hunter 2018), Maximizing benefits and minimizing harm (Hunter 2020)
  - Social responsibility (Junter 2020)
  - Careful consideration of individual and public interests (Hammack 2018)
  - possible negative consequences for the individuals participating in research (Gehner 2016)
  - the importance of the impact research might have on personal relationships, recognising that the implications go beyond those for the individual. They stress the importance of paying particular attention to protecting children and third parties that are not directly involved in a study, especially when researching in smaller communities and ensuring that the benefits of the research are reciprocal for those under study (Gehner 2016)
  - Benefits & Harms must be weighed against each other. Key questions [11]:
    - How can big data be utilized for the common good whilst respecting individual rights and liberties?
    - What are the acceptable trade-offs between individual rights and the common good?
    - How do we determine the thresholds for such trade-offs?

## Research purpose

- - Research integrity (Hunter 2020)
  - Reliability / Bias
    - referred to in (Denecke 2017)
    - referred to as authenticity in (Hunter 2018)
  - (Vitak 2017) misaligned goals and concerns about overreach.
- The communication in social media only becomes valuable for public health after data analysis and interpretation. The interpretative value of the resulting information needs to be verified and corroborated with other means (e.g. medical data, patient interviews/focus groups) [11].

# Privacy

Data privacy is defined as

- ‘freedom from unauthorized intrusion’ [15].
- "freedom from damaging publicity, public scrutiny, secret surveillance, or unauthorized disclosure of one’s personal data or information, as by a government, corporation, or individual"[16]

In particular, the right to internet and privacy issues is enshrined in Article 12 of the Universal Declaration of Human Rights ^[[2]](#footnote-2)^, and Article 17 of the International Treaty Convention on Civil and Political Rights ^[[3]](#footnote-3)^.

What is authorized in social media, is usually defined in the privacy settings, which are frequently refined and reset leaving the user often capitulating under the unsurmountable task to understand them and ensuring the protection of the own data. Often, the service providers secure the right for tracking and reusing the material to their own liberty and advantage.

Past experiences with misuse of data (e.g. Cambridge Analytica) show that there is a need to consider – with each study - need to consider a study’s potential harm to society, as well as to individuals [17]. Likewise, a better delineation is required what of the social media communication is considered public or private, and how such information can be used for research [3–10–18–19].

# Risk of identification

When collected for the purpose of mental health research, illness identification and acting upon this, these data may have implications for personal and professional life, lead to stigmatization or even impact credit or insurance applications [20]. Hence, it crucial to assure data privacy and confidentiality is assured for the collected data. This can be achieved by several means. Firstly only necessary data is collected and identifiable information is excluded. Data access permission policies as well as computer infrastructure should cater for the safe use of data and prevent data loss or disclosure. Reporting should also exclude any identifiable information, e.g. when using quotes or describing patient population, to prevent re-identification.

Rare diseases pose a special risk of re-identification for the patients engaging in online support groups as they often have rather unique combination of characteristics that are reported, such as marital status, age, sex, condition and a 3-digit ZIP code [21].

# Sharing other’s information

A special set of risks in social medial research is related to the possibility of sharing others’ information without their consent and with the risk of their identification through disclosure of personal information.

Sharenting (parent shares news and images of their child on social media) is another example whereby caregivers and parents may disclose personal information of their children with illness and disability [22]. The United Nations Convention on the Rights of the Child and Joel Feinberg’s principle of the child’s right to an open future require that children with illness and disability have the same rights as healthy children to privacy, identity and an open future and that publication of their illness on a social media platform violates these rights.

# Informed consent and public nature of the data

Several authors point out the ***controversy between data manifested public*** by the posts’ authors and the ***need for consent for extraction and processing*** [3–20–23] and it is suggested that “do no harm” generally requires all research participants to be adequately informed about the project, to have consented to the research they are participating in, and to have had their identities protected [7–18–19–24].

On the one hand, it may not be feasible to collect the consent retrospectively from a large number of individuals, and the consent may be implied in terms and conditions of the use of platforms or by public nature of the posts [17–20]. However, still there is a concern about the “informed” nature of this consent and true understanding by the users of all the implications of these Terms of Consent. Unsurprisingly, many patients posting publicly available data on social media are not always comfortable with these data to be used for specific research purposes – especially if they are fearing stigmatization or other disadvantages.

Even if users have consented in the platform settings to the use of their data and the platform’s business model is based on selling such data, the consent cannot really be classified as voluntary but more as ‘induced’ or even ‘coerced’ [7]. In addition, the user terms often change and settings are reset, which complicates the transparent management and administration of user consent over time [25].

Typically, in more ‘traditional’ research, targeted study participants would be approached and asked to consent to participate in a study which is explained to them including the study objectives, the methods, the use of their data, and any potential risks to them. With social media research, where data are gathered from various openly accessible platforms which the users may have used under assumed names or avatars, the consent step is bypassed and thus, the individuals have no knowledge that his or her information is being used for research purposes [26].

Some authors argue that the notion of publicly available data on social media is problematic as the owners may not be intending for the general public to access and see their posts. In a study of representing family lives on Instagram users’ understanding of what is public was found to be diverse and contradictory [3]. A number of users reset their accounts from public to private after being contacted by researchers. Flexible strategies to ethical conduct of research using such data were proposed such as seeking permission from each user or cartoonifying real pictures [3]. However, such flexible approach is only applicable with a very limited amount of data and does not seem feasible with larger scale analysis.

# IRB / REC

It is controversially discussed how SM data should be handled in research and no consensus has been found yet. Consequently, researchers and Ethical Review Boards / Committees are left to their own devices in the development of their own frameworks based on trial and error learning approaches in an environment of rapidly changing and developing methods [7]. The fast development of SM technologies prevents the formation of fixed prescriptive rules and rather suggests the formation of a flexible framework for things to consider and approaches to employee to maintain ethical conduct in public health research using social media data.

The key themes in such approach are (1) privacy, (2) anonymity and confidentiality, (3) authenticity, and (4) the rapidly changing global environment [7].

- participants can be identified either directly or through the internet links related to the websites used, which arouses concerns about the definition of privacy in social media research and the need to evaluate each research study in context and on its own merits [27]
- Deliberative vs institutionalized

## Qualifications and expertise

The traditional way of assuring ethical standards are met by going through research ethics committee may be challenging in the area of social media research. REC members often lack professional or interactional experience, guidelines they could refer to, and most of them would use certain strategies to come up with the decisions for which they lack expertise [28]. Hence, requirements that are not imposed by REC/IRB due to lack of expertise, but have potential ethical implications, should also be considered by SM researchers as much as possible [29]. More so, it is important for REC members to engage and listen to the researchers as their expertise may originate from such engagement [28].

Several authors suggested that dedicated “technology ethics boards” could be convened in universities and other research organizations to educate and advise scientists, research participants, IRBs, and the public (comprised of individuals with expertise in the technology as well as those versed in the ethical, legal, and social implications of data use) [8–29]. Such board should represent a broad range of perspectives (Research, sociology, patients, social media communication, computer scientists / data experts, legal knowledge) to reflect the breadth of perspectives from which Social Media Research is undertaken or to be reviewed [1–29–30].

However, before rigid standards and technology RECs are in place, it is important that over-prudence does not impede social media research with great potential benefit. Some authors argue that a more flexible, nuanced, and bilateral (dialogue between ethics review and researchers) approach should be adopted to social media research ethics to enable use of publicly available social media data without much complication [3–28].

## Reviewers are trained regularly on new developments

IRBs must have access to training on the responsible conduct of social media research to develop the necessary expertise to review it [29].

## Reviewers know the special issues related to social media

A survey among 401 Australian researchers revealed that researchers and HREC members share similar concerns and attitudes about using SM in general and in research [31]. A strong demand for additional support, training, and guidance on SM research ethics was identified, which reflects researchers’ and HREC members’ limited confidence and knowledge of ethical issues in this context and a lack of awareness of available SM-specific ethical guidelines.

- IRB survey in USA [25]

# Required information for reporting the data [32]

## Purpose of the use of the social media data

Are there better methods around / is this the best method?

Is it the right method to meet the goals of the research?

## Choice of the social media data and reasons for choice

Which social media seem to represent patients best? Twitter seems to be the most popular platform used for social media research. The reason for that is not necessarily that this represents best the patients in question but that the data are accessible and there are established processes for doing the research (through an intermediary organization).

## Type of social media content to be applied (text content, images, videos, etc.)

What is the best mix and which methods must be applied to analyse those data?

## Terms of use from the data provider

Each social media platform has its own terms of use and privacy regulations and different level of access to the data. This needs to be considered, justified and accommodated in the research protocol.

## Time period covered by the dataset (incl. periodicity, evolving content)

## Social media user demographics incl. specific population of interest

## Mitigation against any ‘skewing’ / bias

## Generalisability and replicability in the context of the defined purpose

## Principle of secondary use of data

- no planned interactions with the researched community

## Adopted data management approach including data curation

### Named entity recognition (NER) problem in the information retrieval community

- Identifying relevant medicines accounting for phonetic spellings and usage of brand and generic names
- Processing informal text where lay language is used to describe common symptoms or experiences rather than specific illnesses

### Natural language processing (NLP) tools

## Methods and algorithms to be applied and frequency of analysis to be performed

### Quantitative analysis

### Qualitative research techniques

### New and dynamic approaches

NLP, AI, DL, automated or semi-automated grounded theory analyses, etc.

## Demonstrate alignment with applicable data protection legislation and ethical standards

### From data platforms

### In country / countries

## Description / Definition of “public benefit” and “public interest” [33]

# Standard of practice: recommendations

Data processing must respect the applicable data protection legislation (local, where European is not applicable), computer architecture must limit the risk of data loss, and that direct contact of the users (e.g. for life-threatening situations) should be evaluated with legislation experts [23].

# Do's [32–34–35]

## Acknowledge the lack of informed consent or confirmation of diagnosis when interpreting the findings from social media data

## The original ‘poster/blogger’ remains anonymous

## It is the responsibility of the researcher to ensure that any paraphrased quotes included in publications cannot be ‘reverse’ searched to identify the original source

## Take specific care to protect anonymity of contributors or third parties (family, friends, carer, etc.) when reporting sensitive topics or in rare/vulnerable populations

## Consent necessary if there is any risk that anonymity could be at risk

## If an example would help to improve comprehension / clarity, quotes may be paraphrased or re-written

## Investigator transparency (also for third party researchers)

## Comply with the terms of use of the targeted research websites

## Dialogue between researchers and data collectors / Platforms

## Follow good data management practices, consider de- and re-identification; anonymization, coding, aggregation, data sharing, data storage

## Methods and algorithms to be applied and frequency of analysis to be performed are to be documented

Given the variety and potential size of social media data, new and dynamic approaches to existing quantitative and qualitative research techniques should be considered carefully and in sufficient detail to maintain transparency.

## Adhere to 'good research practice' (Hippocratic Oath for data science)

Crowdsourced “Hippocratic Oath for Data Science” (https://datapractices.org/community-principles-on-ethical-data-sharing/): “Ensure that all data practitioners take responsibility for exercising ethical imagination in their work, including considering the implication of what came before and what may come after, and actively working to increase benefit and prevent harm to others.”

## Validation & Generalizability

It needs to be assessed how replicable and generalizable the direct findings of the research are in the context of the defined purpose. In addition, the results must be validated by other means of research (e.g., interviews, focus groups) in order to confirm their relevance for the purpose of assessing a Health Technology.

# Don'ts [14–34–36]

## Websites that require a log-in or password should not be used as this represents a private environment; therefore, individuals would not expect to be observed

## Do not influence the content posted on social media (e.g., posting targeted questions or adding own comments) without consent / transparency

## Do not use Verbatim quotes in reports/publications (risk of re-identification)

Exception: with consent of contributor.

## Don't report any individual demographic data obtained (exception: if necessary to address the research aims)

## Don't use historical text in a non-contextual way

### out of context

### maybe outdated, even from contributor perspective

### historical opt-out or consent difficult / unlikely

## Continuous process

not a 1-time assessment, but reassessment throughout research

# EXCLUDED (as not in scope of review):

- The issues related to the use of social media to recruit hard-to-reach groups (children with cancer) include risk for subject selection bias, privacy rights, protecting identity of participants, data security issues, and access to research [37].
- Information shared by healthcare professionals may represent source of risk for disclosing patients’ potentially identifiable information, such as time frame, patient names, identifiable clinical scenario [38].
- Deleted posts may still represent a source of risk as they remain accessible by analytic software in certain cases. Although this may not be a prevalent issue in general this may still occur and should be taken into consideration when using such data [38].

# Reports & Guidance Summary

**Belmont report 1979**

<https://www.hhs.gov/ohrp/sites/default/files/the-belmont-report-508c_FINAL.pdf>

Belmont report (1979) set out the three basic ethical principles that imply consideration of the following requirements for the conduct of research involving human subjects: informed consent (respect of persons), risk/benefit assessment (benefice), and the selection of subjects of research (justice). Informed consent is a necessary step that has to contain the three elements: information, comprehension and voluntariness. Risks/benefit assessment is carried out by an investigator presented to a review committee and potential participants, who can make informed decision on participation. Finally, there has to be fair procedures and outcomes in the selection of research subjects.

**HMA-EMA Joint Big Data Taskforce – Summary Report (2019)**

<https://www.ema.europa.eu/en/documents/minutes/hma/ema-joint-task-force-big-data-summary-report_en.pdf>

HMA-EMA Joint Big Data Taskforce (2019) does not provide any specific recommendations on the ethical or privacy considerations for using social media data for the conduct of research. However, there is a general recognition that big data and their timely analysis brings value to the regulatory decision-making. It is also anticipated that the volume of social media data will increase and it would be naturally used for research purposes in regulatory space (especially in pharmacovigilance, but other uses merit investigation). The report calls for addressing ethical and privacy issues in access to social media data among other recommendations, but marks it as an immature field.

**””:** *Opportunities exist for m health/wearables to deliver new outcome measures and record lifestyle factors currently not possible to reliably capture via other means, but standards need to be defined if this data is to be used for regulatory submissions. Finally, the most natural application of social media data is for pharmacovigilance and signal detection but further research is required to identify the specific areas where such data will add most value.*

*This situation is replicated over multiple data sets in the big data landscape, particularly real world data and at the far end of the spectrum social media data. However, standardising the data is hard; much of data is unstructured and heterogeneous and this is especially true of social media data which is anticipated to account for much of the data volume increases in the coming years.*

*25 Data access and use To ensure ethical and privacy issues on access to social media data are carefully addressed. • Support the development of guidance on the ethical and legal implications of using social media data. Availability of appropriate guidance.*

**REGULATION (EU) 2016/679 OF THE EUROPEAN PARLIAMENT AND OF THE COUNCIL of 27 April 2016 on the protection of natural persons with regard to the processing of personal data and on the free movement of such data, and repealing Directive 95/46/EC (General Data Protection Regulation)**

<https://eur-lex.europa.eu/legal-content/en/TXT/?uri=CELEX:32016R0679>

In European space, social media research should be conducted in compliance with the General Data Protection Regulation (EU 2016). Among other provisions, the GDPR clearly sets the requirement for informed consent, which could be done by ticking a box on a respective platform/website. The Regulation acknowledges that there may be situations where it is not possible to know the intended purpose of data processing at the time data collection and, therefore, recommends that the subjects should be allowed to give their consent to certain areas of scientific research.

**BERA ETHICAL GUIDELINES (2018) FOR EDUCATIONAL RESEARCH**

<file:///C:/Users/danylan1/Downloads/BERA-Ethical-Guidelines-for-Educational-Research_4thEdn_2018.pdf>

BERA guidelines in 2018 were extended to include the growing field of social media data usage. A few important considerations are raised by BERA in their guidelines.

- They highlight that a given name or identity may not represent a single human (may be a bot, organization, or managed account) and hence, researchers have responsibility to account for these types of users and how their data can or should be processed and shared.
- The authors raise an issue of ambiguity of the informed consent and the extent of public availability of the social media data for the research purposes. They recommend that this has to be addressed in respect to each data source, given its intent and consent process. Researchers are recommended to inform communities about data usage.
- Special attention is given to potential harm that can arise from participation and the obligation to mitigate it immediately.

**Townsend, L., & Wallace**, C. (2016). Social media research: A guide to ethics. Retrieved from <https://www.gla.ac.uk/media/Media_487729_smxx.pdf> [4]
This work was supported by the Economic and Social Research Council [grant number ES/M001628/1] and was carried out at The University of Aberdeen.

**National Committee for Research Ethics in the Social Sciences and the Humanities (NESH)**. (2019) A Guide to Internet Research Ethics. 2^nd^ edition. <https://www.forskningsetikk.no/en/guidelines/social-sciences-humanities-law-and-theology/a-guide-to-internet-research-ethics/>

**British Sociological Association. (2004)**. Statement of ethical practice for the British Sociological Association. Retrieved from <https://www.york.ac.uk/media/abouttheuniversity/governanceandmanagement/governance/ethicscommittee/hssec/documents/BSA%20statement%20of%20ethical%20practice.pdf>

**Association of Internet Researchers** <https://aoir.org/>;

- Internet Research: **Ethical Guidelines 3.0**, Association of Internet Researchers (2019) 🡪 <https://aoir.org/reports/ethics3.pdf>

Markham, Annette, Elizabeth Buchanan, and AoIR Ethics Committee. 2012. “Ethical Decision-Making and Internet Research: Recommendations from the AoIR Ethics Working Committee (Version 2.0).” Association of Internet Researchers (AoIR). <https://aoir.org/reports/ethics2.pdf>.

- Markham & Buchanan, 2012; [2]
- (Norway) A Guide to Internet Research Ethics (Guidelines: RESEARCH ETHICS COMMITTEES | June 2019) <https://ahrecs.com/resources/norway-a-guide-to-internet-research-ethics-guidelines-research-ethics-committees-june-2019/>
- The British Psychological Society. (2017). Ethics guidelines for internet-mediated research (2017).

The British Psychological Society’s (2017) guidelines provide a concise chart for researchers and ethics boards (see Table 1).


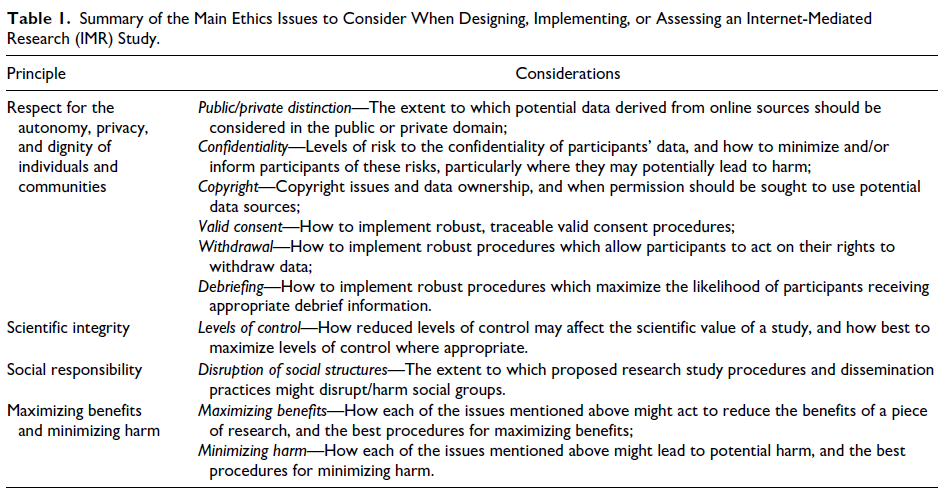


- Kaye, L., Hewson, C., Buchanan, T., Coulson, N., Branley-Bell, D., Fullwood, C., & Devlin, L. (2021). *Ethics guidelines for internet-mediated research*. The British Psychological Society. <https://www.bps.org.uk/news-and-policy/ethics-guidelines-internet-mediated-research> [39]
- British Sociological Association. (2017). Ethics guidelines and collated resources for digital research. <https://www.britsoc.co.uk/media/24309/bsa_statement_of_ethical_practice_annexe.pdf>;
- Council for International Organizations of Medical Sciences. (2016). International ethical guidelines for health-related research involving humans. https://cioms.ch/wp-content/uploads/2017/01/WEB-CIOMS-EthicalGuidelines.pdf; Pagoto, S., & Nebeker, C. (2019). How scientists can take the lead in establishing ethical practices for social media research. Journal of the American Medical Informatics Association, 26, 311–313. <https://doi.org/10.1093/jamia/ocy174>; [29]
- Torous, J., & Nebeker, C. (2017). Navigating ethics in the digital age: Introducing Connected and Open Research Ethics (CORE), a tool for researchers and institutional review boards. Journal of Medical Internet Research, 19(2), e38. <https://doi.org/10.2196/jmir.6793> [40]
- Torous, John, Lyle Ungar, and Ian Barnett. 2019. “Expanding, Augmenting, and Operationalizing Ethical and Regulatory Considerations for Using Social Media Platforms in Research and Health Care.” American Journal of Bioethics 19 (6): 4–6. <https://doi.org/10.1080/15265161.2019.1611278>. [41]

**Social Media Research Guidance; using social media for social research -** A government introduction to social media; from aiding research to the ethical considerations of using such platforms. <https://www.gov.uk/government/publications/social-media-research-guidance-using-social-media-for-social-research>

# References:

1. G. Samuel, G. E. Derrick, & T. van Leeuwen, The Ethics Ecosystem: Personal Ethics, Network Governance and Regulating Actors Governing the Use of Social Media Research Data. *Minerva*, **57** (2019) 317–343. https://doi.org/10.1007/s11024-019-09368-3.

2. A. Markham, E. Buchanan, & AoIR Ethics Committee, *Ethical Decision-Making and Internet Research: Recommendations from the AoIR Ethics Working Committee (Version 2.0)* (Association of Internet Researchers (AoIR), 2012).

3. S. Ravn, A. Barnwell, & B. Barbosa Neves, What Is “Publicly Available Data”? Exploring Blurred Public–Private Boundaries and Ethical Practices Through a Case Study on Instagram. *Journal of Empirical Research on Human Research Ethics*, **15** (2020) 40–45. https://doi.org/10.1177/1556264619850736.

4. L. Townsend & C. Wallace, *Social Media Research: A Guide to Ethics by Townsend and Wallace* (Scotland: University of Aberdeen, 2016).

5. G. Samuel & E. Buchanan, Guest Editorial: Ethical Issues in Social Media Research. *Journal of empirical research on human research ethics : JERHRE*, **15** (2020) 3–11. https://doi.org/10.1177/1556264619901215.

6. S. M. Özkula, The Issue of “Context”: Data, Culture, and Commercial Context in Social Media Ethics. *Journal of empirical research on human research ethics : JERHRE*, **15** (2020) 77–86. https://doi.org/10.1177/1556264619874646.

7. R. F. Hunter, A. Gough, N. O’Kane, G. McKeown, A. Fitzpatrick, T. Walker, M. McKinley, M. Lee, & F. Kee, Ethical Issues in Social Media Research for Public Health. *American Journal of Public Health*, **108** (2018) 343–348. https://doi.org/10.2105/AJPH.2017.304249.

8. S. A. Azer, Social Media Channels in Health Care Research and Rising Ethical Issues. *AMA Journal of Ethics*, **19** (2017) 1061–1069. https://doi.org/10.1001/journalofethics.2017.19.11.peer1-1711.

9. D. Facca, M. J. Smith, J. Shelley, D. Lizotte, & L. Donelle, Exploring the ethical issues in research using digital data collection strategies with minors: A scoping review. *PLOS ONE*, **15** (2020) e0237875. https://doi.org/10.1371/journal.pone.0237875.

10. C. M. Hammack, Ethical Use of Social Media Data: Beyond the Clinical Context. *Hastings Center Report*, **49** (2019) 40–42. https://doi.org/10.1002/hast.979.

11. K. Denecke, An ethical assessment model for digital disease detection technologies. *Life Sciences, Society and Policy*, **13** (2017) 1–11. https://doi.org/10.1186/s40504-017-0062-x.

12. E. Seltzer, J. Goldshear, S. C. Guntuku, D. Grande, D. A. Asch, E. V. Klinger, & R. M. Merchant, Patients’ willingness to share digital health and non-health data for research: a cross-sectional study. *BMC medical informatics and decision making*, **19** (2019) 157. https://doi.org/10.1186/s12911-019-0886-9.

13. O. Rivera-Romero, S. Konstantinidis, K. Denecke, E. Gabarrón, C. Petersen, M. Househ, M. Merolli, & M. Á. Mayer, Ethical Considerations for Participatory Health through Social Media: Healthcare Workforce and Policy Maker Perspectives. *Yearbook of Medical Informatics*, **29** (2020) 071–076. https://doi.org/10.1055/s-0040-1701981.

14. G. Samuel & G. Derrick, Defining ethical standards for the application of digital tools to population health research. *Bulletin of the World Health Organization*, **98** (2020) 239–244. https://doi.org/10.2471/BLT.19.237370.

15. Merian-Webster, Privacy. *Merriam-Webster.com*, (n.d.). https://www.merriam-webster.com/dictionary/privacy (accessed September 20, 2021).

16. Dictionary, Privacy. *www.dictionary.com*, (n.d.). https://www.dictionary.com/browse/privacy (accessed September 20, 2021).

17. Editorial, Cambridge Analytica controversy must spur researchers to update data ethics. *Nature*, **555** (2018) 559–560. https://doi.org/10.1038/d41586-018-03856-4.

18. J. St. John-Matthews, J. Woodley, & L. Robinson, Social media and radiography research: Ethical considerations. *Radiography*, **24** (2018) 96–97. https://doi.org/10.1016/j.radi.2018.03.003.

19. R. A. Hibbin, G. Samuel, & G. E. Derrick, From “a Fair Game” to “a Form of Covert Research”: Research Ethics Committee Members’ Differing Notions of Consent and Potential Risk to Participants Within Social Media Research. *Journal of Empirical Research on Human Research Ethics*, **13** (2018) 149–159. https://doi.org/10.1177/1556264617751510.

20. J. Nicholas, S. Onie, & M. E. Larsen, Ethics and Privacy in Social Media Research for Mental Health. *Current Psychiatry Reports*, **22** (2020). https://doi.org/10.1007/s11920-020-01205-9.

21. J. Gow, C. Moffatt, & J. Blackport, Participation in patient support forums may put rare disease patient data at risk of re-identification. *Orphanet Journal of Rare Diseases*, **15** (2020) 1–12. https://doi.org/10.1186/s13023-020-01497-3.

22. E. Burn, #warriors: sick children, social media and the right to an open future. *Journal of medical ethics*, (2021). https://doi.org/10.1136/medethics-2020-107042.

23. B. Audeh, F. Bellet, M.-N. Beyens, A. Lillo-Le Louët, & C. Bousquet, Use of Social Media for Pharmacovigilance Activities: Key Findings and Recommendations from the Vigi4Med Project. *Drug Safety*, **43** (2020) 835–851. https://doi.org/10.1007/s40264-020-00951-2.

24. J. W. Ayers, T. L. Caputi, C. Nebeker, & M. Dredze, Don’t quote me: reverse identification of research participants in social media studies. *npj Digital Medicine*, **1** (2018). https://doi.org/10.1038/s41746-018-0036-2.

25. J. Vitak, N. Proferes, K. Shilton, & Z. Ashktorab, Ethics Regulation in Social Computing Research: Examining the Role of Institutional Review Boards. *Journal of empirical research on human research ethics : JERHRE*, **12** (2017) 372–382. https://doi.org/10.1177/1556264617725200.

26. M. J. Hammer, Ethical Considerations When Using Social Media for Research. *Oncology Nursing Forum*, **44** (2017) 410–412. https://doi.org/10.1188/17.ONF.410-412.

27. C. Nebeker, J. Harlow, R. E. Giacinto, R. Orozco-Linares, C. S. Bloss, & N. Weibel, Ethical and regulatory challenges of research using pervasive sensing and other emerging technologies: IRB perspectives. *AJOB Empirical Bioethics*, **8** (2017) 266–276. https://doi.org/10.1080/23294515.2017.1403980.

28. C. Sellers, G. Samuel, & G. Derrick, Reasoning “Uncharted Territory”: Notions of Expertise Within Ethics Review Panels Assessing Research Use of Social Media. *Journal of empirical research on human research ethics: JERHRE*, **15** (2020) 28–39. https://doi.org/10.1177/1556264619837088.

29. S. Pagoto & C. Nebeker, How scientists can take the lead in establishing ethical practices for social media research. *Journal of the American Medical Informatics Association*, **26** (2019) 311–313. https://doi.org/10.1093/jamia/ocy174.

30. C. Sellers, G. Samuel, & G. Derrick, Reasoning “Uncharted Territory”: Notions of Expertise Within Ethics Review Panels Assessing Research Use of Social Media. *Journal of empirical research on human research ethics : JERHRE*, **15** (2020) 28–39. https://doi.org/10.1177/1556264619837088.

31. S. Hokke, N. J. Hackworth, S. K. Bennetts, J. M. Nicholson, P. Keyzer, J. Lucke, L. Zion, & S. B. Crawford, Ethical Considerations in Using Social Media to Engage Research Participants: Perspectives of Australian Researchers and Ethics Committee Members. *Journal of Empirical Research on Human Research Ethics*, **15** (2020) 12–27. https://doi.org/10.1177/1556264619854629.

32. S. Brosch, A.-M. de Ferran, V. Newbould, D. Farkas, M. Lengsavath, & P. Tregunno, Establishing a Framework for the Use of Social Media in Pharmacovigilance in Europe. *Drug Safety*, **42** (2019) 921–930. https://doi.org/10.1007/s40264-019-00811-8.

33. L. Bishop, *Big data and data sharing: Ethical issues.* (Manchester: UK Data Service, UK Data Archive., 2017).

34. C. Johnson, H. Kitchen, C. Marshall, J. MacEy, N. V. J. Aldhouse, T. A. Zubeidi, H. C. Pegram, M. Hunter, & S. Knight, Ethical considerations when accessing, analyzing, and reporting social media data. *Quality of Life Research*, **29** (2019) S135. https://doi.org/10.1007/s11136-020-02626-y.

35. M. Metcalf, J. Terkowitz, N. Dasgupty, L. S. Anderson, & G. Powell, Learning by Listening, Digitally. *Value & Outcomes Spotlight (ISPOR)*, **3** (2017) 11–13.

36. N. Hopewell-Kelly, J. Baillie, S. Sivell, E. Harrop, A. Bowyer, S. Taylor, K. Thomas, A. Newman, H. Prout, A. Byrne, M. Taubert, & A. Nelson, Palliative care research centre’s move into social media: constructing a framework for ethical research, a consensus paper. *BMJ supportive & palliative care*, **9** (2019) 219–224. https://doi.org/10.1136/bmjspcare-2015-000889.

37. K. R. Tan, M. K. Killela, & J. Leckey, Ethical Considerations of Social Media to Recruit Caregivers of Children With Cancer. *Nursing research*, **70** (2021) 67–71. https://doi.org/10.1097/NNR.0000000000000473.

38. W. Ahmed, R. Jagsi, T. G. Gutheil, & M. S. Katz, Public disclosure on social media of identifiable patient information by health professionals: Content analysis of twitter data. *Journal of Medical Internet Research*, **22** (2020). https://doi.org/10.2196/19746.

39. L. Kaye, C. Hewson, T. Buchanan, N. Coulson, D. Branley-Bell, C. Fullwood, & L. Devlin, *Ethics guidelines for internet-mediated research* (Leicester, UK: The British Psychological Society, 2021).

40. J. Torous & C. Nebeker, Navigating Ethics in the Digital Age: Introducing Connected and Open Research Ethics (CORE), a Tool for Researchers and Institutional Review Boards. *Journal of Medical Internet Research*, **19** (2017) e38. https://doi.org/10.2196/jmir.6793.

41. J. Torous, L. Ungar, & I. Barnett, Expanding, Augmenting, and Operationalizing Ethical and Regulatory Considerations for Using Social Media Platforms in Research and Health Care. *American Journal of Bioethics*, **19** (2019) 4–6. https://doi.org/10.1080/15265161.2019.1611278.

1. Individuals adopt false identities and establish a false sense of group consensus [↑](#footnote-ref-1)
2. Article 12 “No one shall be subjected to arbitrary interference with his privacy, family, home or correspondence, nor to attacks upon his honour and reputation. Everyone has the right to the protection of the law against such interference or attacks.” [↑](#footnote-ref-2)
3. Article 17 “1. No one shall be subjected to arbitrary or unlawful interference with his privacy, family, home or correspondence, nor to unlawful attacks on his honour and reputation. 2. Everyone has the right to the protection of the law against such interference or attacks.” [↑](#footnote-ref-3)
